# An Online Anomaly Detection Approach for Fault Detection on Fire Alarm Systems

**DOI:** 10.3390/s23104902

**Published:** 2023-05-19

**Authors:** Emanuel Sousa Tomé, Rita P. Ribeiro, Inês Dutra, Arlete Rodrigues

**Affiliations:** 1 Computer Science Department, Faculty of Sciences, University of Porto, 4169-007 Porto, Portugal; emanuel.sousatome@pt.bosch.com (E.S.T.); dutra@fc.up.pt (I.D.); 2 INESC TEC—Institute for Systems and Computer Engineering, Technology and Science, 4200-465 Porto, Portugal; 3 Bosch Security Systems, 3880-728 Ovar, Portugal; arlete.rodrigues@pt.bosch.com; 4 CINTESIS—Center for Health Technology and Services Research, 4200-465 Porto, Portugal

**Keywords:** predictive maintenance, industry 4.0, machine learning, big data, data streams, time series, anomaly detection, fire alarm systems

## Abstract

The early detection of fire is of utmost importance since it is related to devastating threats regarding human lives and economic losses. Unfortunately, fire alarm sensory systems are known to be prone to failures and frequent false alarms, putting people and buildings at risk. In this sense, it is essential to guarantee smoke detectors’ correct functioning. Traditionally, these systems have been subject to periodic maintenance plans, which do not consider the state of the fire alarm sensors and are, therefore, sometimes carried out not when necessary but according to a predefined conservative schedule. Intending to contribute to designing a predictive maintenance plan, we propose an online data-driven anomaly detection of smoke sensors that model the behaviour of these systems over time and detect abnormal patterns that can indicate a potential failure. Our approach was applied to data collected from independent fire alarm sensory systems installed with four customers, from which about three years of data are available. For one of the customers, the obtained results were promising, with a precision score of 1 with no false positives for 3 out of 4 possible faults. Analysis of the remaining customers’ results highlighted possible reasons and potential improvements to address this problem better. These findings can provide valuable insights for future research in this area.

## 1. Introduction

Among the several types of natural and human-made disasters, fire has been one of the most devastating threats in terms of human lives and economic losses [1,2]. The early detection of fires is of major importance, and fire detectors should be guaranteed to work properly and soundly. However, smoke detectors have been reported to failure and are known to be prone to false alarms within specific conditions. Both of these cases pose high risks because a frequent rate of false alarms usually translates into a reduction of human sensitivity to true alarms, thus putting people and buildings at risk. A report from 2021 from the National Fire Protection Association [3] indicated that fire alarms have failed in 16% of home fires in the United States. Consequently, this resulted in significant property damage costs, injured victims, and fire fatalities [4].

Most of the currently implemented maintenance plans for fire alarm systems rely on preventive maintenance, which is prescheduled and does not consider the system’s current state. For instance, the European Standards impose the scheduling of maintenance actions on a regular basis. For example, the EN 54 series mandates that all smoke detectors be tested yearly [5]. This paradigm yields several costs related to possible unnecessary equipment replacement for preventive reasons or even loss of productivity if the interruption of operations and tasks at sites undergoing maintenance is mandatory. Additionally, the maintenance of fire detection systems temporarily renders the system inoperable, with false alarms often occurring during maintenance, which, when interpreted by space users as coming from maintenance, are usually ignored, putting them at risk.

By utilizing sensors, these systems can be upgraded to a new generation that uses data-mining techniques to reduce false alarms and to predict the need for system maintenance. Exploiting data collected on the sensors (e.g., lifetime, reaction to the environment where it is installed, and behaviour over time) can provide valuable information to predict their behaviour, reduce the number of false alarms, and allow failure detection.

In this context, data-driven predictive maintenance (PdM) approaches, particularly anomaly detection approaches, can be applied to inform asset managers about the condition of their fire alarm systems and help them to take informed and timely actions. In particular, anomaly detection aims to identify abnormal data patterns that remarkably deviate from the expected behaviour [6] as a sign of a potential failure. From a data perspective, the goal is to detect anomalies in the behaviour of data over time as described by a set of consecutive unusual data points, i.e., subsequent outliers. There are three main types of anomalies [7]: point (or global), contextual (or conditional), and collective. The goal in PdM is to detect signs of potential failures, which typically configures a contextual outlier, meaning that it is recognized as abnormal behaviour within a temporal time frame.

Most of the approaches available in the literature for anomaly detection have been designed for static datasets [8], i.e., they do not consider the evolving behaviour of the system, which is known to exist in any piece of equipment. Based on our research, we have not yet come across any machine-learning (ML) techniques specifically designed to detect anomalies in smoke detectors’ behaviour. In this context, this work proposes a data-driven anomaly detection approach for anomaly detection of smoke detectors. The method was applied and validated in data obtained from real case studies, from which more than three years of data are available.

This paper is organised as follows. First, an overview of the related work on anomaly detection of sensory systems is given in Section 2. The subsequent section describes the case study on the fire alarm sensory system. Section 4 describes the proposed approach for anomaly detection of smoke detectors. The obtained results are presented in Section 5. Section 6 closes with the main conclusions and outline of further work.

## 2. Related Work

In modern industry, we can find three main maintenance paradigms for a piece of equipment: corrective, preventive, and predictive [9]. Generally, they differ in the maintenance timing and associated costs or savings. Corrective maintenance is the simplest and oldest method. Maintenance occurs only after the breakdown of the equipment. This implies the disruption of the equipment operation and unexpected downtime. Preventive maintenance tries to avoid such downtimes by scheduling regular maintenance actions based on historically known faults. Nevertheless, it involves additional costs of unnecessary maintenance. It may also fail when the failure develops in between maintenance schedules. With the advent of industry 4.0, access to data registered by sensors has become effortless. Leveraging these data to schedule more effective maintenance plans is the ultimate goal of data-driven predictive maintenance (PdM). It comprises a set of techniques that use data analysis tools and machine-learning algorithms to determine when maintenance actions should occur, allowing maintenance to be performed only when it is strictly necessary [10]. Therefore, PdM minimises maintenance costs by extending the useful life of the components [9] and reducing the shut-down time of the equipment. Thus, PdM is increasingly becoming a central cornerstone in industrial applications and systems.

PdM practices are usually grouped into two main tasks: failure prediction and remaining useful life estimation [9,11]. With the former, which can also be called anomaly detection, the main goal is identifying unusual patterns in equipment behaviour to detect potential failures. The latter is concerned with predicting the remaining time of the monitored equipment until the end of its useful life based on its current state and operating conditions. This paper is focused on the first; that is, the anomaly detection of smoke detectors using condition monitoring data. Anomaly detection is a broader problem with a vast number of application domains, such as cybersecurity, predictive maintenance, fault prevention, automation, or e-health [6]. Timely identification of anomalies is essential to tackling underlying problems that, if undetected, may lead to costly consequences [6].

Similar to any data-driven learning technique, data-driven PdM techniques are usually divided into three learning approaches: supervised, unsupervised, and semisupervised. Supervised learning relies on labelling that classifies each instance as normal or abnormal. This usually involves an expert that performs the labelling based on domain knowledge and maintenance reports. However, this approach comes with two main problems: the first is that very few examples of abnormality are expected when compared to the examples of normality, which poses additional difficulties to the learning task [12]. Second, this does not ensure that all possible abnormal behaviours are described in the data for ensuring that the algorithm can learn precisely what constitutes a fault. For this reason, such a context is not often encountered. In fact, most of the data-driven techniques follow either unsupervised or semisupervised approaches. In an unsupervised learning setting, the data have no labels, and the goal is to identify anomalies without any prior knowledge. Nevertheless, in many of the PdM applications, it is possible to leverage the data collected during the normal functioning of a piece of equipment. This configures a semisupervised learning context, where the goal is to model expected (normal) behaviour and identify any deviation from that pattern as an anomaly and thus a signal of a potential failure. Another important aspect of the learning paradigm used in PdM is the ability to update the pattern of normality continuously. Pieces of equipment are known to be affected, both by their number of hours in functioning and by external factors, such as temperature [13]. This change in the functioning pattern does not necessarily lead to a potential failure. Thus, it is very important to resort to methods that allow an online learning setting.

Unsupervised learning techniques can also be found in the technical literature. Liu et al. [14] proposed a data compression approach based on edge computing and an outlier detection approach based on isolation forests. Moreover, they demonstrate that the proposed method outperforms the graph and K-means clustering algorithm. Goh et al. [15] identified which sensor was attacked in a cyber-physical system using a recurrent neural network (RNN) together with a cumulative sum method in data from a replicate of a water treatment plant. Li et al. [16] proposed an unsupervised multivariate anomaly detection method based on generative adversarial networks (GANs) using the long short-term memory recurrent neural network (LSTM-RNN) as the base model. They tested the approach on two real-world datasets, the Secure Water Treatment and the Water Distribution datasets. The approach was proven effective in reporting anomalies caused by various cyber-attacks.

With regards to semisupervised learning techniques, several approaches have been proposed since in many practical anomaly detection applications, only training sets from a single class (the normal one) are available [13]. Examples of traditional ML techniques for semisupervised learning include one-class support vector machines (SVMs), which was applied by Garcia-Font et al. [17] for the detection of attacks in smart city sensor networks. Deep-learning techniques have also been used in a semisupervised learning context. Fiore et al. [18] applied a discriminative restricted Boltzmann machine for network anomaly detection. Luo and Nagarajany [19] applied autoencoders (AEs) for anomaly detection in wireless sensor networks in a fully distributed manner. They could detect spikes and bursts in readings from temperature and humidity sensors.

Regarding fire detection systems, most of the related work in the literature [20] has focused on the use of machine-learning algorithms for the detection of fires to facilitate the firefighting task of mitigating the fire threat, with less focus placed on the detection of the failures of these systems. For example, Bahrepour et al. [21] used feed forward neural networks and Bayesian classifiers to reduce the rate of false fire alarms using a wireless sensor network and concluded that the Bayesian model would be the most suitable for this task. They used a relatively small dataset containing 1400 observations and did not address questions about the behaviour of alarms over time. Iyer et al. [22] used several machine-learning algorithms for forest fire detection. They showed that the classifiers can successfully predict small fires with 85% accuracy but did not achieve the same results for large fires, obtaining only 30% accuracy. In this work, they also showed that incorporating the time variable can be beneficial in predicting fires. More recently, Wu et al. [23] used an adaptive threshold of a feature technique and a deep-learning method for smoke and fire detection from images. They combined attribute extraction with a degree of irregularity and the weighted sum of fire direction values with the deep-learning method using the Caffe framework. The results showed that they can successfully detect fire regions and consequently reduce false alarms.

The above-referred works are meaningful contributions to solving problems mainly related to fire detection, guidance, and alerts to the human groups involved. Our study seeks to characterize fire alarm system equipment over time, providing them with autonomy and intelligence for the self-detection of failures; combines (merges) several heterogeneous types of data; and explores volumes of data not previously studied in this area. Moreover, the data acquisition frequency is also a significant difference from the previous studies that, as they are intended for fire detection, have a much higher frequency. We have at our disposal one point every 15 min, at best. In our case study, we aimed to approach the predictive maintenance problem of fire alarm sensory systems following an online anomaly detection approach based on a semisupervised learning strategy, which is described in the following section.

## 3. Case Study

Fire alarm systems are critical components of a security and safety system of a building since in case of failure, they can cause casualties or severe property damage. A fire alarm system can have multiple devices and components, such as panels, smoke detectors that incorporate air-quality-monitoring sensors, video-based fire detection devices, or sirens.

This work aims to develop a data-driven predictive maintenance approach that issues an alert whenever a smoke detector of a fire system is predicted to suffer a failure when an abnormal pattern is detected. Smoke detectors are life-saving devices whose primary goal is to detect the presence of smoke or fire. Different types of technologies for fire and/or smoke detection are available: photoelectric detectors (also known as optical smoke detectors), ionization detectors [24], microwave radiometers [25], and or image-based detectors [26]. This work is focused on anomaly detection of optical smoke detectors. The readings from the optical sensor are used for this purpose. The optical sensor takes advantage of the light-scattering phenomenon to detect the smoke particles’ size and density within a measuring chamber embedded in the detector. Some smoke detectors can also be equipped with other sensors, such as temperature and chemical sensors. However, to develop the detection of anomalous behaviour in devices only equipped with optical sensors, we only used optical readings in this study.

Two datasets are available: Condition Monitoring (CM) and Remote Alert (RA). The former covers information about the physical building conditions and health status of the fire system components, and a new data point is usually retrieved every 15 min. The latter has information about events (fire, warning, trouble, etc.).

**Figure 1:**
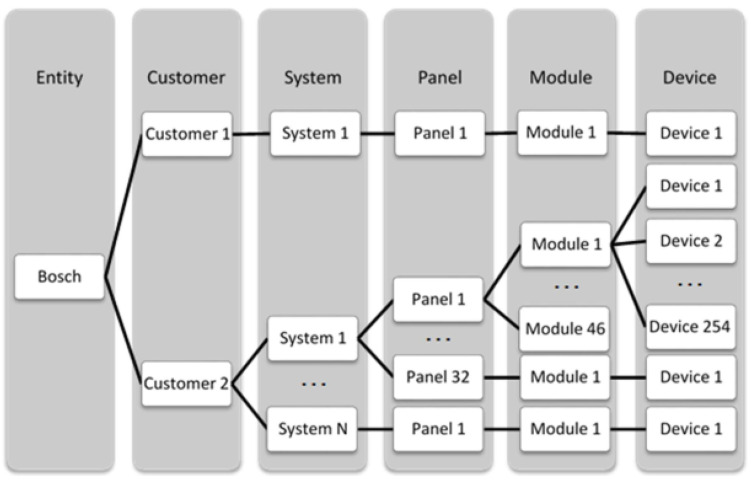
Bosch fire system structure.

Figure 1 presents the general structure of the fire alarm system. For each Bosch customer, having a nonlimited number of systems is possible. Each system can have a maximum of 32 panels, and each panel can have a maximum of 46 modules. Each module can also have loops.

As stated above, fire alarm systems are very complex and life-saving components of a building. Therefore, developing data-driven predictive maintenance approaches is of considerable importance to reducing and preventing malfunctions and system downtime.

## 4. Proposed Approach

The proposed data-driven predictive maintenance approach aims to detect when a smoke detector should be replaced. An online procedure is followed, i.e., the machine learning (ML) model is trained in a predetermined time window and predicts the anomaly scores of the examples in a subsequent forecasting window. If the anomaly score is high, an alarm is triggered. Whenever a new forecasting window data is available, the online learning is repeated by moving the training time window forward.

**Figure 2:**
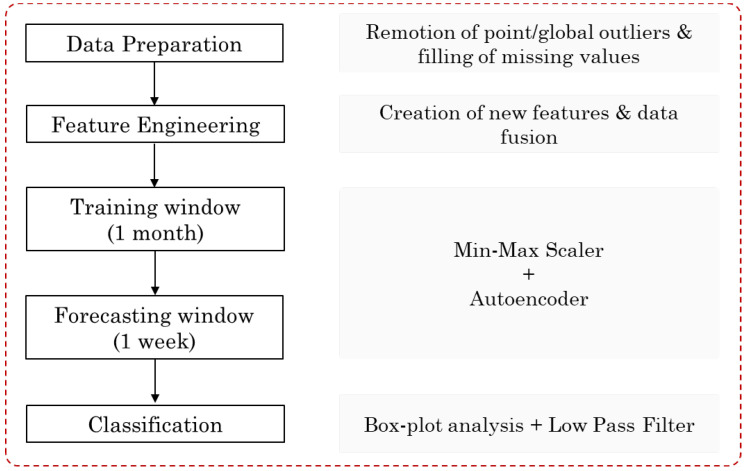
Flowchart of the proposed Data-driven Predictive Maintenance approach.

The approach comprises the following main steps, depicted in Figure 2: data preparation, feature engineering, training an autoencoder with one month of data, using it to forecast the following week, and classifying each example as normal or abnormal. We elaborate on each of these steps next.

### 4.1. Data Pre-Processing

After importing the CM and RA datasets, we performed feature extraction and data wrangling to transform the data into tidy datasets. Each dataset refers to data from a target loop. In this step, a data-cleaning procedure was also considered to impute missing values (e.g., in the device’s serial number) and the possibility of removing outliers from readings.

For the latter, we resorted to the box plot of a daily moving window, i.e., through considering samples of fixed size corresponding to one day, each point is analysed in the context of that moving window. According to Tukey’s box plot definition, if the point is outside the interval $\left[Q_{1}-3\times IQR,Q_{3}+3\times IQR\right]$, where $Q_{1}$ and $Q_{3}$ are the first and third quartiles and IQR is the interquartile range $\left(Q_{3}-Q_{1}\right)$, it is regarded as an extreme outlier and thus can be removed.

As we did not have information regarding the different sensors’ locations, we decided to perform our analysis per loop. The principle is that sensors in the same loop are, in principle, closer to each other. However, each loop can have up to 255 devices, which can lead to situations in which some devices are not physically that close or even exposed to different environments. After the data preparation, feature engineering takes the features, which in this case were extracted from raw data, and aggregates them so that eventually, more useful information is supplied for the machine-learning algorithm [27]. Since the same type of sensor/variable can have different sampling frequencies, the variables were resampled to a common sampling frequency.

The following features were created from optical smoke detector readings in the CM dataset:

- opt1—average of the optical readings (opt1) in the aggregation time interval;
- count_opt1—number of opt1 readings in the aggregated time interval;
- opt1_diff1—first differences of the opt1 variable;
- opt1_kurtosis—kurtosis of the opt1 readings in the aggregation time interval;
- opt1_skewness—skewness of the opt1 readings in the aggregation time interval;
- loop_opt1—average of the opt1 readings of all devices in the loop of the target device, excluding the target device;
- loop_count_opt1—average number of opt1 readings of all devices in the loop of the target device, excluding the target device;
- loop_opt1_diff1—average of the first differences of the opt1 readings of all devices in the loop of the target device, excluding the target device;
- loop_opt1_kurtosis—average of the kurtosis of the opt1 readings of all devices in the loop of the target device, excluding the target device;
- loop_opt1_skewness—average of the skewness of the opt1 readings of all devices in the loop of the target device, excluding the target device.

With regards to the RA dataset, all available states regarding events (such as fire, warning) were aggregated into groups and, for each group, a feature was created with the number of states in the resampling interval as follows:

- fire_state;
- pre_alarm_state;
- fault_state;
- service_state.

**Figure 3:**
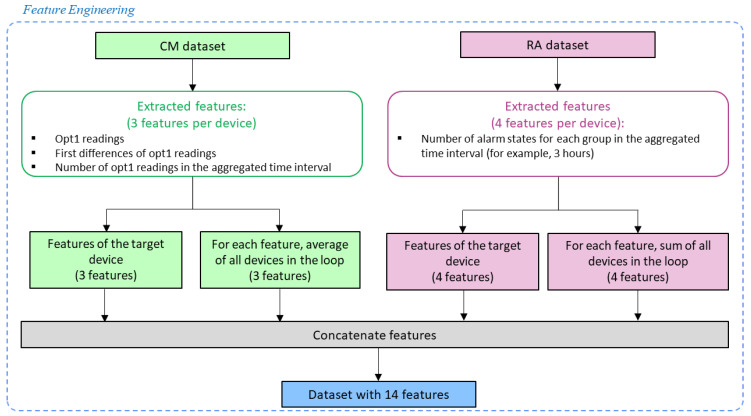
Flowchart of the feature engineering process.

In a similar manner in which the features were extracted from the CM dataset, the features extracted from the RA dataset were created for the target device and for all devices in the loop (excluding the target device). Therefore, four features were extracted from the RA dataset representing the target device and four features representing the loop of the target device. The features representing the loop were obtained by summing all the features of the devices in the loop (except the target device). Figure 3 depicts a flowchart of the feature-engineering stage for the default parameters of the pipeline.

Finally, two additional binary features were also created:

- work_day—1 if it is a weekday, 0 if it is a weekend day.
- day_night—1 if hour ≥8 and hour <20 but 0 otherwise

### 4.2. Online Anomaly Detection

**Figure 4:**
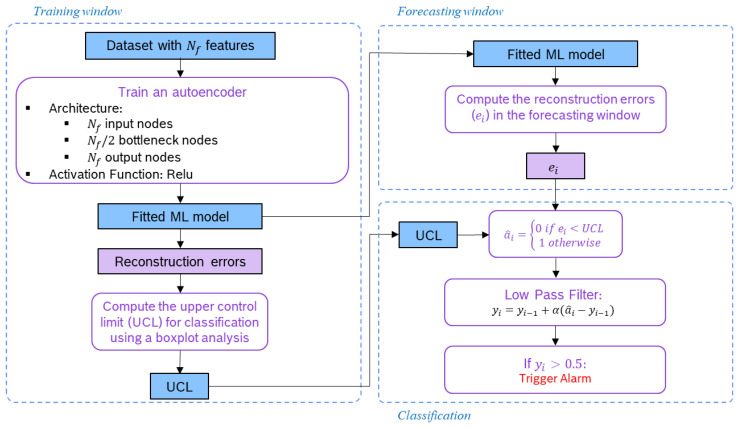
Flowchart of the machine-learning process: training, forecasting, and classification stages.

After the feature engineering stage, a machine-learning (ML) model was fitted using the data from a moving window to capture the evolving normal behaviour of the sensor. We set the training window to 30 days and resorted to the autoencoder (AE) algorithm to build a model that learns to reproduce the input data from that time frame. We should note that all the features were normalised using min–max scaling on the training window. As for the AE architecture, we decided to define only one bottleneck layer composed of the number of nodes equal to half of the input features and activation function Relu. This procedure is schematised on the left part of Figure 4.

The fitted model is then used to forecast the features in the window following the training window, which we set to one week. Then, based on the model’s residuals, each is classified as normal or abnormal following a semisupervised approach. If the model is able to reproduce well the features in the forecasting window, the residuals are considered similar to the ones obtained for the data used for fitting the AE model. Otherwise, if the fitted model cannot reproduce the features in the forecasting window, the residuals are expected to be higher. The latter case indicates a novelty or anomaly, and thus an alarm is triggered.

We implemented this classification process with two approaches: one using the univariate box plot analysis and the other using the multivariate Mahalanobis distance. Regarding the first approach, the mean squared error of the reconstruction error ($e_{i}$) regarding all the features of a given instance was computed using Equation (1),

(1) $$e_{i}=\frac{1}{N}{\left(x_{i}-{\hat{x}}_{i}\right)}^{T}\left(x_{i}-{\hat{x}}_{i}\right)$$

where *N* is the number of features, $x_{i}$ is the input vector with *N* features for timestamp *i*, and ${\hat{x}}_{i}$ is the vector with the model forecast. Next, a box plot analysis was needed to set the upper control limit (UCL). Based on the third quartile ($Q_{3}$) and on the interquartile range (IQR) of the distribution of the mean squared errors obtained for the training window, we used the $UCL=Q_{3}+1.5\times IQR$, as defined by the box plot for high and extreme outlier values. This threshold is used to classify each instance in the forecasting window as normal or abnormal, according to

(2) $${\hat{a}}_{i}=\left\{\begin{array}{c} 0\;\;if\;\;e_{i}<UCL\\ 1\;\;otherwise \end{array}\right.$$

where ${\hat{a}}_{i}$ is the anomaly output prediction obtained based on $e_{i}$ which is the reconstruction error for the instance in timestamp *i*, and UCL is the box plot threshold for identifying high extreme outliers obtained from the distribution of the mean-squared reconstruction errors in the training window. Instances for which the reconstruction error is lower than the UCL are considered normal and predicted as 0. All the others, i.e., those with a reconstruction error equal to or higher than the UCL, are considered abnormal and predicted as 1.

A low-pass filter (LPF) is then used to reduce the number of false alarms. This filter smooths the output of the previous step so that isolated anomaly predictions do not trigger an alarm. Only subsequent anomaly predictions trigger an alarm. The applied LPF is defined as follows

(3) $${\hat{y}}_{i}={\hat{y}}_{i-1}+\alpha \left({\hat{a}}_{i}-{\hat{y}}_{i-1}\right)$$

where ${\hat{y}}_{i}$ is the filter output obtained based on the previous filter output ${\hat{y}}_{i-1}$ and on the anomaly prediction based on the reconstruction error ${\hat{a}}_{i}$, and alpha is the smoothing parameter, also designated as the forgetting factor. The initial value for the recursion is ${\hat{y}}_{0}=0$. If ${\hat{y}}_{i}$ is bigger than 0.5, this indicates that subsequent anomaly predictions have been made, and thus an alert should be triggered. In this case, ${\hat{y}}_{i-1}$ is reset to 0 in the next forecasting window. It should be noted that two slightly different approaches are available and were implemented. Before computing the mean square of the reconstruction errors, the reconstruction errors may or may not be standardised.

Alternatively to the box plot approach, a classification algorithm based on the Mahalanobis distance was also implemented. It follows a similar strategy to the one described above. Nevertheless, the reconstruction error in this case is represented by a vector of size equal to the number of features ($e_{i}$) and the squared Mahalanobis distance for timestamp *i* is computed by as follows:

(4) $$DM_{i}^{2}={\left(e_{i}-\bar{e}\right)}^{T}S^{-1}\left(e_{i}-\bar{e}\right)$$

where $e_{i}=x_{i}-{\hat{x}}_{i}$, $\bar{e}$ is the vector with the average of the residual errors obtained for the training window, and S is the covariance matrix of the residual errors obtained using the reconstruction errors of the training window. The UCL is then defined as the 0.95 quantile obtained for the training window, and Equations (2) and (3) are sequentially applied similarly to the box plot analysis approach described.

Figure 4 presents the flowchart with a full description of these steps for the training, forecasting, and classification stages.

### 4.3. Evaluation

**Figure 5:**
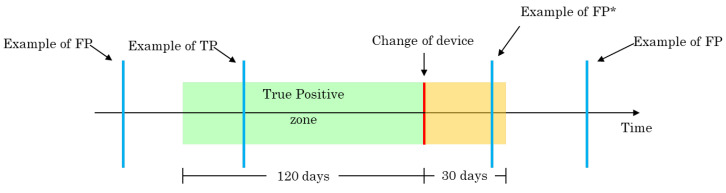
Schematic representation of the evaluation procedure.

To evaluate the proposed approach, the evaluation scheme shown in Figure 5 was used. A description of the alarm outcomes and their conditions defined according to this scheme are as follows:

- True positive (TP): if there is an alert 120 days before the actual change of device;
- False positive (FP): if there is an alert outside the interval defined for TP;
- False negative (FN): if there is no alert in the interval defined for TP;
- False positive* (FP*): if the alarm is in the month after the actual change of the serial.

Multiple alerts distancing less fewer that seven days apart are grouped into a single event. Note that the FP* alarms, i.e., alarms in the yellow zone of Figure 5, are not considered for the computation of the evaluation metrics. This means that if an anomaly (ground truth) does not have an alarm in the TP zone, it is still considered an FN even if there is an alarm in the FP* zone.

For performance estimation, the considered metrics were the following: precision=TP/(TP+FP), recall=TP/(TP+FN), and F1=(2·precision·recall)/(precision+recall).

## 5. Experimental Study

A total of 22 features were considered for the experiments, with 10 representing the target devices, 10 representing the loop where the target device is inserted, and 2 correspond to the binary features work_day and day_night. A total of 11 datasets described by feature subsets were considered with combinations of the 22 available features. The features that compose each dataset are presented in Table 1.

Besides the feature subsets presented in Table 1, six different aggregation times were also considered: 3, 4, 6, 8, 12, and 24 h.

Next, we present the obtained results for a device chosen to demonstrate the approach for all the devices of the same customer where a change of the device took place. Then, we present the obtained results for the other three customers. It should be noted that we used the device change as the ground truth by tracking the serial number. However, a device change can occur not only due to a malfunction but also due to a multitude of factors. For this reason, a high number of FNs were expected.

### 5.1. Illustrative Case

The proposed approach was applied to customer Cust27, for which there were five device changes. Results showed that the approach loses sensitivity to anomalies, i.e., no alarms were triggered for aggregation times higher than 4 h. For this reason, only results for time aggregations of 3 and 4 h are presented. Moreover, no outliers were removed in the preprocessing step, as this did not improve the overall results. The number of TPs, FPs, and FNs obtained for case 1 from customer Cust27 and the amount of time between the first alert and the actual change of device for different time aggregations, feature sets, and classification approaches are presented in Table 2. For this particular case, the box plot without standardisation of the reconstruction errors presents the worst results with a higher number of FPs and FNs for some of the feature sets and a generally shorter anticipation time of the device change. For the other classification approaches, similar results were obtained. In general, the anticipation time was about 100 days, and except for $FS_{1}$, $FS_{2}$, $FS_{4}$, $FS_{7}$, $FS_{8}$, and $FS_{9}$, no FPs or FNs were obtained.

Regarding the time aggregation, similar results were obtained for 3 and 4 h of aggregation time. For this reason, only the results obtained for three hours of aggregation time are presented.

Customer Cust27 also had three more cases in which there was a device change (one with two changes in the evaluated time frame). Except for one of the device changes, all were predicted in timely fashion by the proposed approach.

Table 3 presents the obtained metrics of all the cases of device change for customer Cust27 for the 11 datasets and for the three classification approaches considered: box plot analysis without standardisation of the reconstruction errors, box plot analysis with standardisation of the reconstruction errors, and Mahalanobis distance. Datasets $FS_{1}$ to $FS_{3}$ presented a high number of FPs for all the classification approaches. The datasets $FS_{4}$ to $FS_{6}$ generally showed higher F1-score across all the classification approaches. The remaining feature sets did not exhibit uniform behaviour across the three classification approaches. For instance, $FS_{10}$ had an F1-score of 0.750 for the Mahalanobis distance and for the box plot with standardisation of the reconstruction errors but an F1-score of 0.444 when the box plot without standardisation of the residuals was used for classification.

Ultimately, the features work_day and day_night did not improve the performance. A similar situation occurred regarding the RA features (fire_state, pre_alarm_state, fault_state, and service_state), as they were afterwards found to be redundant. In fact, some of the alarms/states in the RA were triggered by sensors’ readings when reaching specific thresholds.

### 5.2. Results for Other Customers

**Figure 6:**
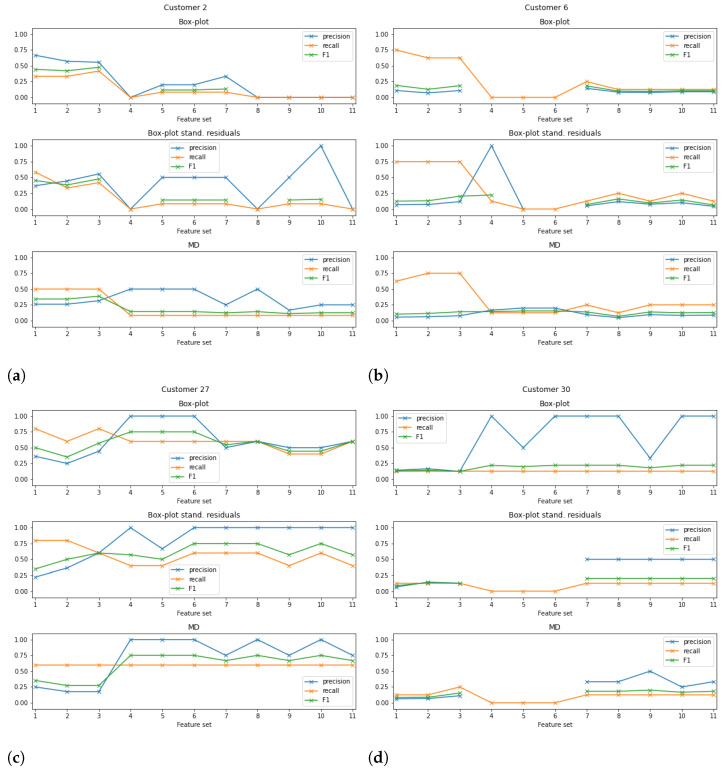
Performance results obtained for customers (**a**) Cust2, (**b**) Cust6, (**c**) Cust27, and (**d**) Cust30. Results present the precision, recall, and F1-score obtained with the 11 feature subsets used for classifying training window of 30 days and box plot analysis without standardisation of the reconstruction errors; box plot analysis with standardisation of the reconstruction errors; and Mahalanobis distance (MD) with the threshold computed for the 0.99 quantile.

The results obtained for customer Cust27 are very interesting and promising. However, how the approach performs with data from other customers should also be evaluated. For that purpose, three systems from three different customers with more device changes were selected. Thus, customers Cust2, Cust6, and Cust30 were chosen since they had 12, 8, and 8 device changes for the analysed period, respectively. Figure 6 presents the precision, recall, and F1-score obtained for customers Cust2, Cust6, Cust27, and Cust30 for the 11 datasets considered.

The first conclusion that can be taken from the analysis of the plots is that there is no classification approach or dataset with better metrics across all the customers. For instance, while for customer Cust27, the datasets with the worst performance were $FS_{1}$ to $FS_{3}$, for customer Cust2, these were the feature sets with the best performance metrics when using the box plot analysis without standardisation of the reconstruction errors (cf. Figure 6a).

Moreover, for some datasets in customers Cust6 and Cust30, the approach did not trigger any alarms. This may be explained by the fact that a device change does not necessarily mean that the replaced device had a malfunction. Indeed, in some cases significant differences in the sensor readings were not spotted for any of the considered features. Cases such as these are not expected to be flagged by the anomaly detection approach since any variability/change is present in the data. As already mentioned, device changes such as these may not be related to a malfunction, or the used/available features may not be sensitive to the anomaly. For this reason, for further work and development, the exploration of other features, such as energy consumption or "FallTime" of the optical sensor, should be carried out [4]. Other meta-features may also be of interest [28].

A training window of 90 days was also considered to evaluate the sensitivity of the performance metrics to this hyperparameter. However, similar results were obtained.

## 6. Conclusions

Fire detectors, such as smoke detectors, should be guaranteed to work properly and soundly since their inefficient behaviour or failure can lead to damaging, and sometimes irreversible, consequences to society and the economy, such as causalities or property damage. False alarms and periodic maintenance of the fire detection system are the two cost factors associated with the fire detection system in a given building.

In this context, this paper described a practical application of an online data-driven predictive maintenance approach for anomaly detection of smoke detectors. The online procedure involves training an autoencoder in a predetermined time window and predicting anomaly scores of the examples in a subsequent forecasting window. If the anomaly score is high, an alarm is triggered. Whenever the date from a new forecasting window are available, the online learning is repeated by moving the training time window forward.

The proposed approach was applied to over three years of data obtained from independent fire alarm systems installed with four customers. Without further information on possible failures, the tracking of the device change was used as the ground truth. However, a device change may not be related to abnormal behaviour. For this reason, a high number of FNs were obtained. A total of 22 features were considered, extracted from continuous monitoring and event data. The obtained performance values for different features and classification approaches are presented in this paper.

The method proved robust for one of the customers (Cust27), detecting three out of four device changes without any FPs (precision of 1). However, the results were not of the same quality for the remaining customers. It was impossible to obtain a combination of feature set and classification approach with consistent performance across all the customers. Therefore, tuning the method for each specific case will always be necessary. Indeed, this is an expected outcome in real-case applications [29]. Each fire alarm system was located in a different building with different environmental conditions and activities. Thus, in the future, considering the geolocation and/or the building core activity may help to improve the results obtained.

The less than satisfactory performance for some customers may also be related to a lack of information on the considered features regarding the abnormal behaviour of the device. Therefore, further developments to improve this approach should explore other domain-specific features, such as the energy consumption of “FallTime” [4]. Moreover, experimenting with more meta-features may also be of interest [28]. Finally, only parametric analysis of input features and classification approaches were considered in this work. Other autoencoder architectures and ML models, such as principal component analysis, can also be tested in the future.

## Figures and Tables

**Table 1:** Composition of the 11 feature sets. Check marks (✓) indicate included features.

	Features																					
Features Subset	opt1	count_opt1	opt1_diff1	opt1_diff24h	opt1_kurtosis	opt1_skewness	fire_state	pre_alarm_state	fault_state	service_state	work_day	day_night	loop_opt1	loop_count_opt1	loop_opt1_diff1	loop_opt1_diff24h	loop_opt1_kurtosis	loop_opt1_skewness	loop_fire_state	loop_pre_alarm_state	loop_fault_state	loop_service_state
$FS_{1}$	✓	✓	✓				✓	✓	✓	✓			✓	✓	✓				✓	✓	✓	✓
$FS_{2}$	✓	✓	✓										✓	✓	✓							
$FS_{3}$	✓	✓	✓		✓	✓							✓	✓	✓		✓	✓				
$FS_{4}$		✓	✓		✓	✓								✓	✓		✓	✓				
$FS_{5}$		✓	✓		✓									✓	✓		✓					
$FS_{6}$		✓	✓		✓				✓					✓	✓		✓					
$FS_{7}$		✓	✓	✓	✓				✓					✓	✓	✓	✓					
$FS_{8}$		✓	✓	✓	✓									✓	✓	✓	✓					
$FS_{9}$		✓	✓	✓	✓						✓	✓		✓	✓	✓	✓					
$FS_{10}$		✓	✓	✓	✓						✓			✓	✓	✓	✓					
$FS_{11}$		✓	✓	✓	✓							✓		✓	✓	✓	✓					

**Table 2:** Number of TPs, FPs, and FNs and time before the device change of the first alarm triggered by the proposed approach for case 1 from customer Cust27.

Classifier:		Box Plot No Stand. Residuals					Box Plot Stand. Residuals					Mahalanobis Distance				
**Time**	**Feat.**					**Anticip.**					**Anticip.**					**Anticip.**
**Aggreg.**	**Subset**	**TP**	**FP**	**FN**	FP*	**(Days)**	**TP**	**FP**	**FN**	FP*	**(Days)**	**TP**	**FP**	**FN**	FP*	**(Days)**
3 h	$FS_{1}$	1	0	0	0	65	1	1	0	0	67	1	0	0	0	64
	$FS_{2}$	1	0	0	0	65	1	0	0	0	65	1	1	0	0	64
	$FS_{3}$	1	0	0	0	64	1	0	0	0	99	1	0	0	0	101
	$FS_{4}$	1	0	0	0	63	1	0	0	0	101	1	0	0	0	100
	$FS_{5}$	1	0	0	0	98	1	0	0	0	101	1	0	0	0	97
	$FS_{6}$	1	0	0	0	63	1	0	0	0	101	1	0	0	0	98
	$FS_{7}$	1	0	0	0	88	1	0	0	0	101	1	0	0	0	64
	$FS_{8}$	1	1	0	0	88	1	0	0	0	100	1	0	0	0	100
	$FS_{9}$	0	0	1	0	-	1	0	0	0	101	1	0	0	0	100
	$FS_{10}$	1	0	0	0	88	1	0	0	0	100	1	0	0	0	101
	$FS_{11}$	1	0	0	0	96	1	0	0	0	101	1	0	0	0	98
4 h	$FS_{1}$	1	0	0	0	64	1	0	0	0	66	1	0	0	0	64
	$FS_{2}$	1	0	0	0	64	1	0	0	0	63	1	0	0	0	64
	$FS_{3}$	1	0	0	0	63	1	0	0	0	100	1	0	0	0	99
	$FS_{4}$	0	0	1	0	-	1	0	0	0	100	1	0	0	0	99
	$FS_{5}$	1	0	0	0	100	1	0	0	0	100	1	0	0	0	99
	$FS_{6}$	1	0	0	0	63	1	0	0	0	100	1	0	0	0	100
	$FS_{7}$	1	1	0	0	64	1	0	0	0	100	1	0	0	0	99
	$FS_{8}$	0	0	1	0	-	1	0	0	0	98	1	0	0	0	99
	$FS_{9}$	1	0	0	0	88	1	0	0	0	100	1	0	0	0	99
	$FS_{10}$	1	0	0	0	88	1	0	0	0	100	1	0	0	0	99
	$FS_{11}$	1	0	0	0	88	1	0	0	0	100	1	0	0	0	99

**Table 3:** Performance results for customer Cust27. The best F1-Score is highlighted in bold for each classification approach and feature set.

Classifier	Metric	Feature Subset										
		${\mathrm{FS}}_{1}$	${\mathrm{FS}}_{2}$	${\mathrm{FS}}_{3}$	${\mathrm{FS}}_{4}$	${\mathrm{FS}}_{5}$	${\mathrm{FS}}_{6}$	${\mathrm{FS}}_{7}$	${\mathrm{FS}}_{8}$	${\mathrm{FS}}_{9}$	${\mathrm{FS}}_{10}$	${\mathrm{FS}}_{11}$
Box plot	TP	4	3	4	**3**	**3**	**3**	3	3	2	2	3
no stand.	FP	7	9	5	**0**	**0**	**0**	3	2	2	2	2
residuals	FN	1	2	1	**2**	**2**	**2**	2	2	3	3	2
	FP*	1	1	2	**0**	**0**	**0**	0	0	0	0	0
	Precision	0.364	0.250	0.444	**1.000**	**1.000**	**1.000**	0.500	0.60	0.500	0.500	0.600
	Recall	0.800	0.600	0.800	**0.600**	**0.600**	**0.600**	0.600	0.600	0.400	0.400	0.600
	F1	0.500	0.353	0.571	**0.750**	**0.750**	**0.750**	0.545	0.600	0.444	0.444	0.600
Box plot	TP	3	3	3	**3**	**3**	**3**	3	**3**	3	**3**	3
stand.	FP	9	14	14	**0**	**0**	**0**	1	**0**	1	**0**	1
residuals	FN	2	2	2	**2**	**2**	**2**	2	**2**	2	**2**	2
	FP*	0	0	0	**0**	**0**	**0**	0	**0**	0	**0**	0
	Precision	0.250	0.176	0.176	**1.000**	**1.000**	**1.000**	0.750	**1.000**	0.750	**1.000**	0.750
	Recall	0.600	0.600	0.600	**0.600**	**0.600**	**0.600**	0.600	**0.600**	0.600	**0.600**	0.600
	F1	0.353	0.273	0.273	**0.750**	**0.750**	**0.750**	0.667	**0.750**	0.667	**0.750**	0.667
Mahalanobis	TP	4	4	3	2	2	**3**	**3**	**3**	2	**3**	2
Distance	FP	14	7	2	0	1	**0**	**0**	**0**	0	**0**	0
	FN	1	1	2	3	3	**2**	**2**	**2**	3	**2**	3
	FP*	0	0	0	0	1	**0**	**0**	**0**	0	**0**	0
	Precision	0.222	0.364	0.600	1.000	0.667	**1.000**	**1.000**	**1.000**	1.000	**1.000**	1.000
	Recall	0.800	0.800	0.600	0.400	0.400	**0.600**	**0.600**	**0.600**	0.400	**0.600**	0.400
	F1	0.348	0.500	0.600	0.571	0.500	**0.750**	**0.750**	**0.750**	0.571	**0.750**	0.571

## Data Availability

Not applicable.

## References

[1] Baek J., Alhindi T.J., Jeong Y.S., Jeong M.K., Seo S., Kang J., Shim W., Heo Y. (2021). Real-time fire detection system based on dynamic time warping of multichannel sensor networks. Fire Saf. J..

[2] Kong S.G., Jin D., Li S., Kim H. (2016). Fast fire flame detection in surveillance video using logistic regression and temporal smoothing. Fire Saf. J..

[3] Ahrens M. (2021). Smoke Alarms in US Home Fires (NFPA ®) Key Findings.

[4] Tambe A., Nambi A., Marathe S. Is your smoke detector working properly? Robust fault tolerance approaches for smoke detectors. Proceedings of the 19th Annual International Conference on Mobile Systems, Applications, and Services.

[5] (2004). Fire Detection and Fire Alarm Systems—Part 14: Guidelines for Planning, Design, Installation, Commissioning, Use and Maintenance.

[6] Erhan L., Ndubuaku M., Di Mauro M., Song W., Chen M., Fortino G., Bagdasar O., Liotta A. (2021). Smart anomaly detection in sensor systems: A multi-perspective review. Inf. Fusion.

[7] Chandola V., Banerjee A., Kumar V. (2009). Anomaly Detection: A Survey. ACM Comput. Surv..

[8] Salehi M., Rashidi L. (2018). A Survey on Anomaly detection in Evolving Data [with Application to Forest Fire Risk Prediction]. SIGKDD Explor. Newsl..

[9] Davari N., Veloso B., Costa G.d.A., Pereira P.M., Ribeiro R.P., Gama J. (2021). A survey on data-driven predictive maintenance for the railway industry. Sensors.

[10] Carvalho T.P., Soares F.A., Vita R., Francisco R.d.P., Basto J.P., Alcalá S.G. (2019). A systematic literature review of machine learning methods applied to predictive maintenance. Comput. Ind. Eng..

[11] Wang Q., Zheng S., Farahat A., Serita S., Gupta C. Remaining useful life estimation using functional data analysis. Proceedings of the 2019 IEEE International Conference on Prognostics and Health Management, ICPHM 2019.

[12] Branco P., Torgo L., Ribeiro R.P. (2016). A Survey of Predictive Modeling on Imbalanced Domains. ACM Comput. Surv..

[13] Ribeiro R.P., Pereira P., Gama J. (2016). Sequential anomalies: A study in the Railway Industry. Mach. Learn..

[14] Liu D., Zhen H., Kong D., Chen X., Zhang L., Yuan M., Wang H. (2021). Sensors Anomaly Detection of Industrial Internet of Things Based on Isolated Forest Algorithm and Data Compression. Sci. Program..

[15] Goh J., Adepu S., Tan M., Lee Z.S. Anomaly detection in cyber physical systems using recurrent neural networks. Proceedings of the IEEE International Symposium on High Assurance Systems Engineering.

[16] Li D., Chen D., Jin B., Shi L., Goh J., Ng S.K. In Proceedings of the MAD-GAN: Multivariate Anomaly Detection for Time Series Data with Generative Adversarial Networks BT—Artificial Neural Networks and Machine Learning—ICANN 2019: Text and Time Series.

[17] Garcia-Font V., Garrigues C., Rifà-Pous H. (2016). A comparative study of anomaly detection techniques for smart city wireless sensor networks. Sensors.

[18] Fiore U., Palmieri F., Castiglione A., De Santis A. (2013). Network anomaly detection with the restricted Boltzmann machine. Neurocomputing.

[19] Luo T., Nagarajany S.G. Distributed anomaly detection using autoencoder neural networks in WSN for IoT. Proceedings of the IEEE International Conference on Communications.

[20] Abid F. (2021). A Survey of Machine Learning Algorithms Based Forest Fires Prediction and Detection Systems. Fire Technol..

[21] Bahrepour M., Meratnia N., Havinga P.J. (2009). Use of ai techniques for residential fire detection in wireless sensor networks. CEUR Workshop Proc..

[22] Iyer V., Iyengar S.S., Nandan P., Garmiela R.M., Mandalika M.B.S. Machine Learning and Dataming Algorithms for Predicting Accidental Small Forest Fires. Proceedings of the SENSORCOMM 2011: The Fifth International Conference on Sensor Technologies and Application.

[23] Wu X., Lu X., Leung H. An Adaptive Threshold Deep Learning Method for Fire and Smoke Detection. Proceedings of the 2017 IEEE International Conference on Systems, Man, and Cybernetics (SMC).

[24] Fonollosa J., Solórzano A., Marco S. (2018). Chemical sensor systems and associated algorithms for fire detection: A review. Sensors.

[25] Alimenti F., Roselli L., Bonafoni S. (2016). Microwave radiometers for fire detection in trains: Theory and feasibility study. Sensors.

[26] Li P., Zhao W. (2020). Image fire detection algorithms based on convolutional neural networks. Case Stud. Therm. Eng..

[27] Zheng A., Casari A. (2018). Feature Engineering for Machine Learning.

[28] Rivolli A., Garcia L.P., Soares C., Vanschoren J., de Carvalho A.C. (2022). Meta-features for meta-learning. Knowl. Based Syst..

[29] Huyen C. (2022). Designing Machine Learning Systems.

